# Baron Larrey on Wounds of the Throat

**Published:** 1831-04-01

**Authors:** 


### On Wounds of the Throat. By Baron Larrey.

[Clinique Chirurgicale.]

*Case* 1. M. Arrighi (now Duke of Padua, and then aid-de-camp to General Berthier) received a musket-ball in his neck, at the seige of Acre, by which the external carotid artery was cut across, near to the place where it is given off from the internal, and as it enters the parotid gland. The gush of blood from both apertures of the wound attracted the attention of the artillery men, and one of them instantly pushed a finger into each opening, and thus arrested the flow of blood. Baron Larrey was immediately called amidst a shower of shot and shells. He applied pressure and maintained it carefully for some days, by which means, and without any ligature, life was preserved, and all hæmorrhage prevented.

*Case* 2. After the battle of Waterloo, the Baron had an opportunity of seeing a young English soldier who had had the left external carotid artery *partially* opened. The hæmorrhage was alarming; but the English surgeon cut down on the aperture, and tied the artery both below and above the wound. The patient entirely recovered.

*Case* 3. Henry Gabon, of the Swiss Guard, was brought into the Hôpital de la Garde, on the 21st November, 1828, immediately after receiving a sabre-wound, while fighting a duel, in the upper part and right side of the neck. When the Baron arrived the man was nearly dead from hæmorrhage and suffocation. The wound was laid bare, while an assistant made pressure on the line of the artery, and then the Baron enlarged the orifice, and discovered that the superior thyroid artery was wounded, as also the external carotid itself. A cellular pouch had formed behind the thyroid gland, (which was goitrous,) filled with clotted blood, and which was pressing on the trachea. The removal of these clots was followed by a jet of arterial blood. The Baron was unable to seize the vessels from which the blood issued, and therefore laid bare the trunk of the common carotid, and passed a ligature round it. He was not a little surprized to find this artery no larger than the radial artery at the wrist. This was attributable to the great loss of blood. The great source of hæmorrhage was thus cut off; but some vessel still continued to supply blood at the upper part of the wound. This vessel was fortunately seized by the forceps and secured. The wound was then cleaned and dressed. The breathing continued difficult, and the lips deadly pale. For two or three days it was doubtful whether this man would rally; but eventually he recovered.

*Case* 4. A grenadier of the army of Egypt was wounded by a bayonet, the broken point of which remained, for six weeks, deep in the left side of the pharynx, behind the arch of the palate. The man had entirely lost his voice. The Baron, with great difficulty, seized the foreign body and extracted it. The voice was instantly restored. The iron had pressed on the laryngeal branch of the par vagum.

*Case* 5. A subaltern officer of the Guards was brought into the hospital on the 7th of June, 1824, presenting a wound in the neck, on the right of the larynx, so small as to be scarcely perceptible. There was great ecchymosis and tumefaction of the whole anterior region of the neck, with deep-seated pain in the chest. Voice and speech were gone—the respiration exceedingly difficult, as well as deglutition. He informed Baron Larrey, by writing, that this wound was made by a small sword. Venesection was repeatedly employed, together with cupping and leechings, which gave some relief. On the sixth day, however, he was menaced with suffocation, and his face was blue and bloated. The Baron found him apparently in the agonies of death. In this crisis he determined on tracheotomy. He made an incision through the integuments of some length, and then perforated the space between the thyroid and cricoid cartilages. An immense explosion of air was the immediate consequence, together with the expulsion of several clots of blood. Respiration succeeded, and considerable relief was the result. A paroxysm of suffocation, however, soon after occurred, owing to the obstruction of the orifice in the air-passage, and a tube was quickly inserted. Relief was again obtained; but thirst was intolerable, and the unhappy patient was unable to swallow. In this dilemma, a tube was, with great difficulty, passed into the stomach, and fluids introduced into that organ. The thirst was moderated; but he could not bear the presence of the hollow bougie, and tore it out himself. He lingered in dreadful agony, till four o'clock the next morning, when he expired.

On dissection, an abscess was found in front of the three superior cervical vertebræ, (which were denuded,) the size of a hen's egg, and which had pressed so much the parietes of the pharynx against the cricoid cartilage and upper part of the trachea, that respiration could not be carried on through the aperture that was made by the knife. A purulent infiltration had also penetrated down into the chest through the cellular membrane.

The Baron, in his remarks on this case, does not allude to the possibility of life being saved, if the opening had been made lower down in the trachea, instead of the place which he pitched on for the operation. In all cases where tracheotomy is deemed necessary, the lower down the operation is performed, the more difficult it is—but the greater is the chance of success, for the obvious reason that we are thus the more likely to get below the obstruction.

*Case* 6. General Murat (afterwards King of Naples) received, at the battle of Aboukir, a musket-shot, which traversed the neck, from side to side, wounded the root of the tongue, and carried away a portion of the epiglottis. The Baron was on the spot, and rendered immediate assistance. The first phenomenon which he observed, was the discharge of the dismembered portion of the epiglottis, followed by a considerable expectoration of frothy blood. The General was harrassed for some days with painful cough, loss of voice, &c. The Baron cleared the orifices of the wound both at its entrance and exit, and then introduced an elastic tube into the œsophagus, for the purpose of introducing liquid nourishment and drink into the stomach. This was necessary, as there was no proper valve to prevent the ingress of substances into the trachea. In the course of 18 days, however, the parts had so accommodated themselves to the loss of a portion of the epiglottis, that this officer was able to swallow with little or no inconvenience.

*Case* 7. In this case, which was that of a soldier in Egypt, who was wounded by a musket-ball on the 21st of March, 1801, the whole of the epiglottis was carried away. The poor fellow was devoured by thirst, but could not drink, and harrassed with incessant cough. In this dreadful state he continued four days, without any relief. When Baron Larrey saw him he was in the most piteous and dangerous condition. The Baron was enabled to pass a gum-elastic tube down the œsophagus, and through this to introduce liquids into the stomach. By a long and assiduous perseverance in this measure, the life of the soldier was saved, and Nature supplied the place of the epiglottis by a contrivance of her own.

Two other cases, nearly similar, are related by the Baron, but the foregoing are, we think, sufficient for the elucidation of the present subject. In another short article, we shall give some cases of wounds of the œsophagus, from the same ample source of information.

